# Blind Quality Assessment of Iris Images Acquired in Visible Light for Biometric Recognition †

**DOI:** 10.3390/s20051308

**Published:** 2020-02-28

**Authors:** Mohsen Jenadeleh, Marius Pedersen, Dietmar Saupe

**Affiliations:** 1 Department of Computer and Information Science, University of Konstanz, 78457 Konstanz, Germany; dietmar.saupe@uni-konstanz.de; 2 Department of Computer Science, Norwegian University of Science and Technology, N-2802 Gjøvik, Norway; marius.pedersen@ntnu.no

**Keywords:** biometric recognition, visible light iris images, image quality assessment, image covariates, quality filtering

## Abstract

Image quality is a key issue affecting the performance of biometric systems. Ensuring the quality of iris images acquired in unconstrained imaging conditions in visible light poses many challenges to iris recognition systems. Poor-quality iris images increase the false rejection rate and decrease the performance of the systems by quality filtering. Methods that can accurately predict iris image quality can improve the efficiency of quality-control protocols in iris recognition systems. We propose a fast blind/no-reference metric for predicting iris image quality. The proposed metric is based on statistical features of the sign and the magnitude of local image intensities. The experiments, conducted with a reference iris recognition system and three datasets of iris images acquired in visible light, showed that the quality of iris images strongly affects the recognition performance and is highly correlated with the iris matching scores. Rejecting poor-quality iris images improved the performance of the iris recognition system. In addition, we analyzed the effect of iris image quality on the accuracy of the iris segmentation module in the iris recognition system.

## 1. Introduction

The stability of iris patterns over the human lifespan and their uniqueness was first noticed in 1987 [1]. Since then, biometric iris recognition has been extensively investigated for accurate and automatic personal identification and authentication [2]. Most commercial iris recognition systems use near-infrared (NIR) images. However, due to the popularity of smartphones and similar handheld devices with digital cameras, iris recognition systems using images taken in visible light have recently been developed [3,4,5].

Image quality is a key factor affecting the performance of iris recognition systems [6,7,8]. In the biometric recognition literature, a biometric quality measure is a covariate that is measurable, influences performance, and is actionable [9,10,11]. Quality measurement can include subject and image covariates. Subject covariates are attributes of a person, which may be properties of subjects such as eyelid occlusion, glare, iris deformation, or wearing of glasses. Image covariates depend on sensor and acquisition conditions, such as focus, noise, resolution, compression artifacts, and illumination effects. In this work, we develop a real-time quality measure for image covariates as an actionable quality score, e.g., to decide whether an input iris image sample should be enrolled into a dataset or rejected and a new sample should be captured.

The performance of an iris recognition system in visible light suffers from all of the image quality factors mentioned above. To overcome this problem, some researchers have considered image quality in different ways for iris recognition systems [12,13,14,15,16,17]. However, these systems fall short in two ways:

- The considered image covariates and distortions are limited. Only distortions are taken into account that are often seen, such as Gaussian blur, noise, motion blur, and defocus. However, authentic iris images, especially those taken by handheld devices, may additionally suffer from other types of distortion.
- Typically, quality assessment is applied to accurately segmented iris images. However, image distortion also affects the performance of the segmentation module of iris recognition systems. Thus, poor image quality can lead to poorly segmented irises and increase in the false rejection rate.

In this paper, we propose a general-purpose and fast image quality method that aims to assess the distortion of iris images acquired in unconstrained environments. This method can be used for real-time quality prediction of iris images to rapidly filter image samples with poor quality. Iris images with insufficient quality could lead to high dissimilarity scores for matching pairs and increase the false rejection rate of an iris recognition system. We investigate the effect of iris image quality on the recognition performance of a reference iris recognition system for three challenging iris image datasets acquired in visible light.

This paper is an extended version of our conference paper [18] and mostly a part of the Ph.D. thesis of the first author [19]. The remainder of the paper is organized as follows: Section 2 surveys the literature on iris image quality assessment and iris recognition systems. Section 3 presents the proposed metric for iris image quality assessment. In Section 4, experiments are conducted to study the effect of image quality on the accuracy of iris segmentation. In Section 5, the improvements achieved by filtering poor-quality iris images are discussed using three performance measures on three large iris image datasets acquired in visible light. The paper concludes with suggestions for future research in Section 6.

## 2. Related Work

In this section, we review the literature on iris image quality assessment, followed by a brief overview of some state-of-the-art iris recognition systems.

Recently, research has been reported to improve the performance of iris recognition systems by considering image quality, but with certain limitations. In some studies, image quality has been examined by considering only certain quality factors, such as sharpness [20], out-of-focus [21], and JPEG compression [22]. These metrics alone cannot be expected to produce reliable quality assessments of authentic in-the-wild iris images.

In some other work, iris image quality metrics are applied after segmentation of the iris. In [23], the result of the iris segmentation module is used to form a quality score. Happold et al. [24] proposed a method for predicting the iris matching scores of an iris image pair based on their quality features. They calculated these features for precisely segmented iris images. They labeled a dataset of iris image pairs with the corresponding matching scores. They trained their method for predicting the matching score of an image pair based on their quality features. Therefore, these methods cannot be used to measure iris image quality in the iris recognition system pipeline before segmentation.

Several metrics for iris image quality were developed based on a fusion of several quality measures of image and subject covariates. The authors of [25,26] combined quality measures relating to motion blur, angular deviation, occlusion, and defocus into an overall quality value of an input iris image. These quality metrics were developed for NIR-based images and compared to traditional NIR-controlled iris image acquisition settings. However, images in visible light and under uncontrolled lighting conditions result in notorious differences in the appearance of the acquired images [3]. Therefore, this method may not be used directly to evaluate the quality of iris images in visible light. Li et al. [27] proposed a method for predicting an iris matching score based on iris quality factors such as motion blur, illumination, off-angle, occlusions, and dilation. This method requires segmented irises to compute some of these quality factors (dilation and occlusions).

The authors of [10] used combined subject and image covariates, such as the degree of defocusing, occlusion, reflection, and illumination, to form an overall quality score. They focused on the evaluation of iris images after iris segmentation, which allows the systems to process images of poor and good quality in the acquisition phase. They considered only a few image covariates for quality estimation.

Proença [3] proposed a metric for the quality assessment of iris images taken in visible light. This metric measures six image quality attributes such as focus score, off-angle score, motion score, occlusion score, iris pigmentation level, and pupil dilation. Then, the impact of image quality on feature matching was analyzed. The results showed a significant performance improvement of the iris recognition system by avoiding low-quality images. However, this method requires precisely segmented iris images, and only the motion-blur score is combined with some quality factors related to the subject’s covariates.

The authors in [12] proposed an approach that automatically selects the regions of an iris image with the most distinguishably changing patterns between the reference iris image and the distorted version to compute the feature. The measured occlusion and dilation are combined to form a total image quality score to study the correlation between iris image quality and iris recognition accuracy.

In the approach of [28], the image quality is assessed locally, based on a fusion schema at the pixel level using a Gaussian mixture model, which gives a probabilistic measure of the quality of local regions of the iris image. The local quality measure is used to detect the poorly segmented pixels and remove them from the fusion process of a sequence of iris images.

Recently, many image quality methods have been proposed for perceptual quality assessment of natural images [29,30,31,32,33,34,35]. Some of these models use statistics of completed local binary patterns (CLBP) as a part of their feature vectors. In [33], joint statistics of local binary patterns (LBP) and CLBP patterns produced quality-aware features, and a regression function was trained to map the feature space to the perceived quality scores. In [32], features based on several local image descriptors such as CLBP, local configuration patterns (LCP), and local phase quantization (LPQ) were extracted, and then a support vector regressor was used to predict the quality scores. These models are trained to predict the perceptual quality of natural images. Liu et al. [36,37] studied some of these methods for filtering low-quality iris images. This study showed inconsistencies for the predicted quality, e.g., removing more low-quality images did not always increase the performance of the iris recognition system. In addition, they removed the low-quality images for each subject separately. Therefore, the filtered images do not have the same range of quality, and there is no global quality-filtering threshold.

In summary, some of the methods for iris quality assessment, such as [25,26], are proposed for NIR images, and only a few types of distortion are considered. Some other quality metrics, like those in [3,23,24], require a segmented iris image to calculate their quality features. They also take limited distortion types into account and are not expected to work well for quality assessment of authentic iris images taken in visible light in arbitrary environmental conditions. Iris recognition systems based on authentic images will broaden the scope of iris recognition systems, and require more research to develop robust metrics for quality assessment of authentically distorted iris images.

Since we used an iris recognition system as a reference system in this paper, in the following, we briefly review some state-of-the-art iris recognition systems.

The fast iris recognition (FIRE) system for images acquired by mobile phones in visible light was proposed by Galdi et al. [38]. It is based on the combination of three classifiers by exploiting iris color and texture information. Raja et al. [39] proposed a recognition system for iris images captured in visible light. This method extracts deep sparse features from image blocks and the whole iris image in different color channels to form the feature vector for an input iris image. Minaee et al. [40] proposed an iris feature extraction method based on textural and scattering transform features. The principal component analysis (PCA) technique is used to reduce the extracted feature dimension.

Recently, OSIRIS version 4.1, an open-source iris detection system, was proposed by Othman et al. [41]. This system follows the classic Daugman method [42] with some improvements in segmentation, normalization, coding, and matching modules. For iris and pupil segmentation, the Viterbi algorithm is used for optimal contour detection. For normalization, a non-circular iris normalization is performed using the coarse contours detected by the Viterbi algorithm. The coding module is based on 2-D Gabor filters, which are calculated in different scales and resolutions. Finally, the matching module calculates the global dissimilarity score between two iris codes using the Hamming distance. We used this system as a reference iris recognition system.

## 3. Proposed Method

In this section, we present our fast and general-purpose method for assessing the quality of iris images acquired in visible light.

Earlier works on iris recognition [42,43] employed block-based operations to obtain iris features. Therefore, we can infer that the most distinctive information in the iris pattern comes from the local patterns of an iris image rather than from global features. Local binary patterns (LBP) and their derivatives have been successfully used in many pattern recognition applications, including texture classification [44,45,46], image retrieval [47,48], object recognition [49,50], action recognition [51,52], and biometric recognition [53,54,55,56].

Most of the LBP-based biometric recognition methods use statistical analysis of local patterns for their feature extraction. Wu et al. [29] showed that image distortions could change the statistics of LBPs. They then examined the statistics of the LBPs to suggest an index for evaluating natural image quality. However, this index does not accurately predict image quality for some common image distortions, such as Gaussian blur and impulse noise.

In the proposed differential sign–magnitude statistics index (DSMI), sign and magnitude patterns are first derived. Then, the statistical characteristics of these patterns are analyzed for their sensitivity to iris image distortion. Statistical features of specific coincidence patterns with high sensitivity to image distortion are identified. A weighted nonlinear mapping is applied to the features to form the iris image quality score. This metric takes advantage of the observation that low-quality iris images have fewer of these patterns compared with those in high-quality iris images.

### 3.1. Proposed Quality Metric

Our iris image quality metric uses statistical features extracted from patterns of signs and magnitudes of local intensity differences. Then, certain locally weighted statistics of specific sign–magnitude coincidence patterns are used to define the quality score. Guo et al. [46] suggested a completed local binary pattern (CLBP) to represent the local difference information that is missed in the LBP representation of an image [57]. We investigate how common distortions in iris images could alter the statistics of the CLBP. Then, a quality metric based on a specific coincidence of sign and magnitude patterns of the CLBP is proposed.

In CLBP, a local grayscale image patch is represented by its central pixel, and the local differences are given by $d_{p}$ = $x_{p}-x_{c}$, where $x_{c}=I\left(c\right)$ is the gray value of the central pixel of the given patch and $x_{p}$ is the gray value of a pixel in the neighborhood. A local difference $d_{p}$ can be decomposed into two components, its sign and its magnitude. These signs and magnitudes of local differences are combined into corresponding patterns, CLBP-S and CLBP-M, as follows.

Let C={(i,j)|i=0,⋯,M−1,j=0,⋯,N−1} be the set of pixels of a normalized grayscale image *I* of *N* pixels width by *M* pixels height. For a given pixel c∈C, let $x_{c}$ and $x_{p},p=0,\cdots ,P-1,$ denote the gray values of the center pixel *c* and the *P* points on a circle of radius *R* about $x_{c}$. For example, suppose the coordinates of $x_{c}$ are (0,0); then, the coordinates of $x_{p}$ are (Rcos(2πp/P),Rsin(2πp/P)). The grayscale value $x_{p}$ is estimated by interpolation if its coordinates do not coincide with the center of a pixel. Then, the CLBP-S patterns are defined by

(1) $${\mathrm{CLBP}-S}_{P,R}\left(c\right)=\sum_{p=0}^{P-1}b_{p}\cdot 2^{p},\;b_{p}=\{\begin{array}{cccc} \; & 1 & \; & x_{p}\geq x_{c}\\ \; & 0 & \; & \mathrm{otherwise} \end{array}.$$

The CLBP-S operator generates the same code as that of the original LBP operator. The CLBP magnitude patterns are defined similarly by

(2) $${\mathrm{CLBP}-M}_{P,R}\left(c\right)=\sum_{p=0}^{P-1}b_{p}\cdot 2^{p},\;b_{p}=\{\begin{array}{cccc} \; & 1 & \; & m_{p}\geq z\\ \; & 0 & \; & \mathrm{otherwise}, \end{array}$$

where $m_{p}=\left|x_{p}-x_{c}\right|$ is the magnitude of the local difference $d_{p}$. Furthermore, the threshold value *z* is the average local difference in the *P*-neighborhoods of all center pixels together, i.e.,

(3) $$z=\frac{1}{|C|P}\sum_{c\in C}\sum_{p=0}^{P-1}\left|x_{p}-x_{c}\right|.$$

For each pixel c∈C, we consider the *P*-bit binary representation of the sums in Equations (1) and (2) as binary codes of $\mathrm{CLBP}-S_{P,R}$ and $\mathrm{CLBP}-M_{P,R}$. Using these binary representations, we define rotation invariant indices or patterns for CLBP-S and CLBP-M in a manner similar to that proposed by Ojala et al. [57] for LBP codes. Equation (4) gives the rotation invariant indices of CLBP-S,

(4) $$\mathrm{CLBP}-S_{P,R}^{riu2}\left(c\right)=G\left(\mathrm{CLBP}-S_{P,R}\left(c\right)\right)=G\left(\sum_{p=0}^{P-1}b_{p}2^{p}\right)=\{\begin{array}{cc} \sum_{p=0}^{P-1}b_{p} & U(\sum_{p=0}^{P-1}b_{p}2^{p})\leq 2\\ P+1 & \mathrm{otherwise} \end{array}.$$

Here, *U* gives the number of bit changes (0 to 1 or 1 to 0) of the *P*-bit binary representation of a number (including circular shift),

$$\;U\left(\sum_{p=0}^{P-1}b_{p}2^{p}\right)=\sum_{p=0}^{P-1}\left|b_{p}-b_{\;\mathrm{mod}\;(p+1,P)}\right|.$$

Similarly, Equation (5) gives the uniform rotation invariant patterns of CLBP-M.

(5) $${\mathrm{CLBP}-M}_{P,R}^{riu2}\left(c\right)=G\left({\mathrm{CLBP}-M}_{P,R}\left(c\right)\right)).$$

Note that these indices, ${\mathrm{CLBP}-S}_{P,R}^{riu2}$ and ${\mathrm{CLBP}-M}_{P,R}^{riu2}$, range over the set {0,…,P+1}. The first indices from 0 up to *P* correspond to local sign and magnitude patterns with only, at most, two bit changes and, thus, denote uniform local patterns. All non-uniform patterns are assigned to the remaining index P+1.

${\mathrm{CLBP}-S}_{P,R}^{riu2}$ generates fewer codes than the basic CLBP-S. It carries less textural information by simplifying the local structure. ${\mathrm{CLBP}-M}_{P,R}^{riu2}$ provides a compact representation of textural information derived from local magnitude patterns.

**Figure 1:**
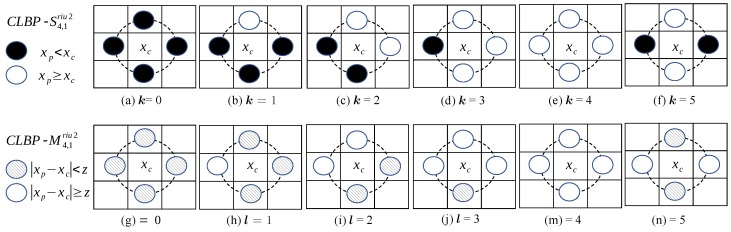
The patterns in the upper row correspond to $\mathrm{CLBP}-S_{4,1}^{riu2}$, which compares the gray value of the central pixel of a patch ($x_{c}$) with the gray values of its four neighbors ($x_{p}$). The black and white disks denote smaller and greater values than those of the central pixel value, respectively. In the lower row, $\mathrm{CLBP}-M_{4,1}^{riu2}$ compares the absolute values of the differences of the gray values of the central pixel and its neighbors (|$x_{c}-x_{p}|$) with the threshold *z* from Equation (3). The hatched and white disks denote smaller and greater absolute values than those of the threshold, respectively. Note that the patterns are rotation invariant. Thus, in the case of P=4 shown here, the patterns for k,l=1,2,3,5 may be rotated by multiples of 90 degrees without changing the values of $\mathrm{CLBP}-S_{4,1}^{riu2}$ and $\mathrm{CLBP}-M_{4,1}^{riu2}$.

For an illustration for the case of P=4 neighbors at distance R=1 from the central pixel of a patch, we provide Figure 1. We obtain six indices *k* and *l* for sign and magnitude patterns, corresponding to five rotation invariant uniform patterns (k,l=0,…,4) and one index (k,l=5) that represents all non-uniform patterns.

Finally, the local indices for sign and magnitude have to be combined to give a quality indicator for an iris image as a whole. We first join the two types of indices into a set of bitmaps $V_{k,l}\left(c\right)$, indexed by k,l,

(6) $$V_{k,l}\left(c\right)=\{\begin{array}{cc} 1 & \mathrm{CLBP}-S_{P,R}^{riu2}\left(c\right)=k\;and\;\mathrm{CLBP}-M_{P,R}^{riu2}\left(c\right)=l\\ 0 & \mathrm{otherwise} \end{array}.$$

For each pair k,l of indices, we form a weighted sum of $V_{k,l}\left(c\right)$ over all pixels *c*, which is nonlinearly scaled to the unit interval by $r\left(x\right)=1-e^{-ax}$ as follows:

(7) $$Q_{k,l}=r\left(\frac{1}{\left|C\right|}\sum_{c\in C}\frac{V_{k,l}\left(c\right)}{{\hat{\sigma }}^{2}\left(c\right)+\delta^{2}}\right).$$

Here, ${\hat{\sigma }}^{2}\left(c\right)$ is the local variance of the *P*-neighboring pixels of the center pixel *c*, and $\delta^{2}$ is a small constant value to prevent division by zero. The parameters $\delta^{2}$ and *a* are empirically set to 0.00025 and 0.01, respectively.

In Equation (7), the normalization by the local variance emphasizes local minima and maxima, and normalizing the scores to the range [0,1) is only for ease of interpretation of the quality scores. The value of $Q_{k,l}$ is considered as an image quality score derived from the sign pattern *k* and the magnitude pattern *l*. In our experiments, we used four neighbors (P=4) with unit distance (R=1) from the central pixel *c* of a local patch.

Our experiments showed that $Q_{k,l}$ with the specific coincidence of the sign pattern k=0 and magnitude pattern l=0 has a high correlation with iris image quality. Therefore, we used $Q_{0,0}$ as our proposed DSMI quality score. We had summarized the proposed DSMI metric in our conference paper [18], considering, however, only the selected coincidence sign–magnitude patterns.

### 3.2. Empirical Justification

Inspired by Wu et al. [29], we examine the distinctiveness of each pattern of $\mathrm{CLBP}-S_{4,1}^{riu2}$, which coincides with patterns of $\mathrm{CLBP}-M_{4,1}^{riu2}$ for separating high-quality iris images from distorted versions. To that end, we generated an artificially distorted iris image dataset from 600 pristine high-quality references taken from the Warsaw-BioBase-Smartphone-Iris v1.0 [4], UTIRIS [58], and $GC^{2}$ multi-modal [36] datasets. A total of 3 to 12 samples per eye from 75 individuals were selected. This dataset was used only to justify our choice of specific sign–magnitude patterns and also to investigate how filtering out the low-quality iris images using the DSMI metric could affect the performance of the segmentation module of the reference iris recognition system. The reference iris images have no content-dependent deformations such as eyelid occlusion, and were selected from individuals with high, medium, and low degrees of iris pigmentation. The irises of all of these reference iris images were segmented accurately by the reference iris recognition system.

Five common image distortions with different levels and multiple distortions were used to distort the reference iris images. These distortions are Gaussian blur (GB), motion blur (MB), white Gaussian noise (WGN), salt and pepper noise (IN), and overexposure (OE). The parameters of each function and the number of the distorted versions of each reference image are listed in Table 1. In addition to the individual types of distortions, we generated multiple distorted iris images (GB+WGN). First, we distorted the images with GB and then with WGN. Since GB tends to occur during the acquisition phase due to the different working conditions of the image sensors, we applied it first. WGN is a noise model that can be used to mimic the effects of random processes, such as sensor noise due to poor illumination and thermal noise in the imaging device. For simplicity, the recommendation of [59] was followed, and WGN was introduced in the end.

**Figure 2:**
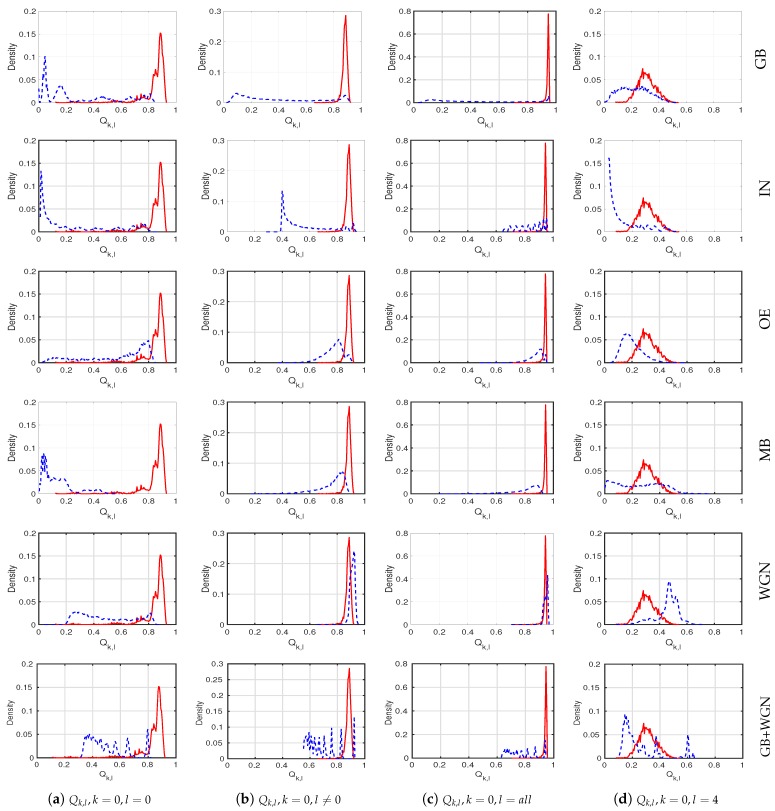
The solid red lines show the distributions of the quality scores of the high-quality iris images, and the dotted blue lines show the distributions for the distorted versions with different distortion types, which are shown on the right side of each row. The quality scores $Q_{k,l}$ are formed based on four different coincidences of sign (*k*) and magnitude (*l*) patterns, shown at the bottom of each column. The first column shows the histograms of the quality score $Q_{0,0}$, and the second, third, and fourth columns show the histograms of the coincidence patterns $Q_{0,l}$ with l≠0, l=all, and l=4.

To analyze the discrimination power of the scores $Q_{k,l}$ for separating the high-quality reference images from their distorted versions, we show the distributions of the corresponding scores $Q_{k,l}$ for some selected combinations of *k* and *l* in Figure 2. Visual inspection clearly shows that the coincidence of sign–magnitude patterns with k=0 and l=0 gives the greatest discrimination power. The predicted quality scores for the reference iris images are mostly between 0.8 and 1, and the scores for the distorted versions are mostly less than 0.8. Therefore, we chose this coincidence pattern to form our DSMI quality metric (DSMI = $Q_{0,0}$).

## 4. Iris Segmentation Accuracy

The performance of iris segmentation in a classical iris recognition system has a significant impact on the overall performance. In this section, we analyze how image distortions affect the performance of the segmentation module and how quality filtering could improve the segmentation.

Most of the state-of-the-art iris recognition systems for iris imaging acquired in visible light, such as FIRE [38], Raja et al. [39], and OSIRIS, version 4.1 [41], can be used as reference iris recognition systems. We have chosen OSIRIS version 4.1 because (1) OSIRIS is an open-source iris recognition system that facilitates reproducible experiments, (2) it shows high recognition performance [41], and (3) it was used as the reference iris recognition system in some recent biometric recognition studies [4,60,61,62,63,64]. The segmentation module of OSIRIS version 4.1 uses the Viterbi algorithm to detect the iris and pupil contours [65]. The outputs are contours of the iris, which represent the inner boundary between the pupil and iris and the outer boundary between the iris and sclera, resulting in a binary mask for the iris.

For our experiments, we used the artificially distorted dataset from the previous section, which is summarized in Table 1. We segmented all iris images using the OSIRIS segmentation module. The mask of the segmented iris of each reference image was taken as the ground truth for comparison with the segmentation results for the distorted versions. The iris segmentation error is computed by the fraction of mislabeled pixels,

$$e=\frac{1}{\left|C\right|}\sum_{c\in C}T\left(c\right)⊕M\left(c\right),$$

where |C| is the cardinality of the pixel set *C* of an iris image, and *T* and *M* represent the ground truth and the generated iris masks, respectively. The symbol ⊕ represents the exclusive OR operation to identify the segmentation error. If the error *e* was below the threshold 0.05, the iris segmentation was assumed to be correct. The threshold value was set manually by the authors.

**Figure 3:**
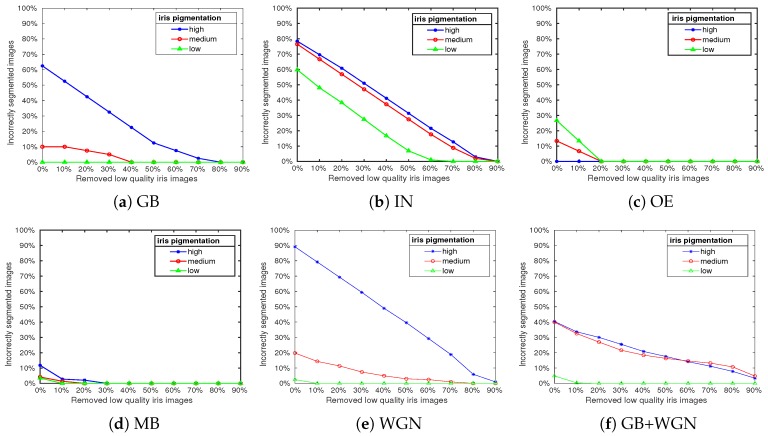
The segmentation performance of the reference iris recognition system is shown for segmenting iris images with high, medium, and low pigmentation, and distorted in different ways. The fraction of incorrectly segmented images is plotted versus the percentage of filtered low-quality images, based on the differential sign–magnitude statistics index (DSMI) metric.

In Figure 3, we show the fractions of incorrectly segmented irises for the different types of distortion and for low, medium, and high degrees of iris pigmentation. The fractions are given as functions of the percentage of low-quality images that were filtered out using the proposed DSMI quality metric.

The results shown indicate a clear correlation between the DSMI quality of iris images and segmentation accuracy. Therefore, filtering out poor-quality images before segmentation will improve the performance by reducing the number of incorrectly segmented images, as indicated by the negative slopes of the plots.

In summary, the experiments performed in this section show that the accuracy of the segmentation module varies for iris images with different pigmentations and different distortions. Highly pigmented iris images present a greater challenge for the reference iris recognition system, while the system is more robust for the segmentation of low-pigmented iris images. However, filtering out poor-quality iris images using the proposed DSMI metric increases the accuracy of iris segmentation.

## 5. Experimental Results

In this section, we investigate to what extent filtering out poor-quality iris images with the proposed quality metric improves the performance of the reference iris recognition system. We also compare our DSMI quality metric with the BRISQUE [66] and WAV1 [67] image quality metrics. BRISQUE uses statistical features extracted from pixel intensities to train a support vector machine for predicting image quality. Pertuz et al. [67] compared 15 metrics to estimate the blur of an image. In their study, WAV1 performed better than the others. WAV1 uses statistical properties of the discrete wavelet transform coefficients. Since blur is a common distortion of iris images taken by handheld imaging devices such as smartphones, we also compare our method with the WAV1 metric. Our experiments were conducted on three large authentic iris image datasets acquired in visible light.

### 5.1. Iris Image Datasets

There are many iris image datasets recorded with near-infrared cameras such as CASIA V4 [68], CASIA-Iris-Mobile-V1 [69], IIT Delhi [70], and ND CrossSensor Iris 2013 [71]. However, there are just a few iris image datasets acquired in visible light. Four are widely used in iris recognition research: UTIRIS [58], UBIRIS [72], MICHE [73], and VISOB [74].

An optometric framework in a controlled environment was used for capturing the irises of the UTIRIS dataset, resulting in high-quality iris images. UBIRIS iris images were taken from moving subjects and at different distances, resulting in more heterogeneous images compared to UTIRIS. Nevertheless, the pictures have good quality, better than the expected quality of iris images captured by handheld devices. The MICHE and VISOB datasets are challenging datasets for iris recognition systems, including images with varying degrees of iris pigmentation and eye make-up. In addition, the quality of the images is impaired by lack of focus, gaze deviations, specular reflections, eye occlusion, different lighting conditions, and motion blur.

Instead, we chose three datasets of the $GC^{2}$ multi-modal biometric dataset [36] because they contain authentically distorted iris images typically seen when capturing iris images with handheld devices such as smartphones. In addition, the iris images were taken from many subjects with different handheld cameras in uncontrolled environments at different distances. Iris pigmentation varied, from European subjects with bright iris textures to Asian subjects with very dark iris textures. In addition to the various authentic distortions corresponding to the image covariates, the iris images are subject to a variety of quality losses related to the subject’s covariates, such as gaze deviation, off-angle, reflections, eye closure, and make-up. Also, the datasets contain 12–15 iris images of varying quality per eye and person, which is useful for studying the effect of quality filtering. The iris images have more than 30 different resolutions.

- The first dataset of $GC^{2}$, REFLEX, was taken with a Canon D700 camera using a Canon EF 100 mm f/2.8 L macro lens (18 megapixels). It contains 1422 irises of 48 subjects. A total of 12 to 15 samples were taken per eye (left and right).
- The second dataset, LFC, contains iris images taken by a light field camera. The LFC dataset contains 1454 iris images from the right and left eyes of 49 subjects. For each eye, 13 to 15 samples were taken.
- The third dataset, PHONE, was taken by a smartphone (Google Nexus 5, 8 megapixel camera). It contains 1379 iris images from the right and left eyes of 50 subjects, and 12 to 15 samples were taken per eye.

**Figure 4:**
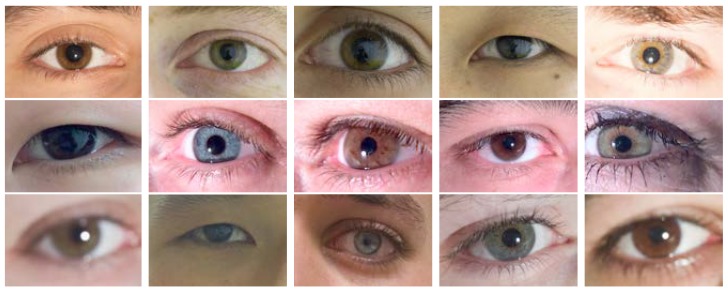
Some iris image samples with high, medium, and low pigmentation from the multi-modal biometric dataset $GC^{2}$ [36]. The first, second, and third rows show some images from the REFLEX, LFC, and PHONE datasets, respectively.

**Figure 5:**
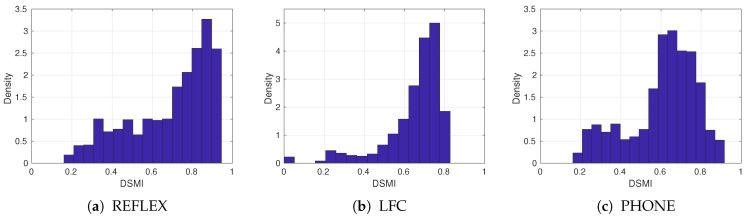
Normalized histograms of the quality scores according to the DSMI metric on three test iris datasets.

We compare an iris image with all iris images from the same dataset. Table 2 summarizes these datasets and shows the number of matching and non-matching iris pairs. Figure 4 shows some samples from these datasets, and Figure 5 shows the histograms of the quality scores of the datasets, estimated by the proposed DSMI metric.

### 5.2. Iris Recognition Performance Analysis

To evaluate the performance improvement of iris recognition achieved by quality filtering using an image quality metric, we used three performance methods, namely the Daugman’s decidability index [75], the area under the receiver operating characteristic curves (AUC), and the equal error rates (EER). We compared the performance of three image quality metrics when used for quality filtering. Given a threshold for a metric, we rejected those images that exhibited a quality lower than the threshold. The thresholds for each of the three metrics were chosen such that 1/4, 1/2, and 3/4 of the images were rejected. In our experiments, OSIRIS version 4.1 was used as a reference iris recognition system.

#### 5.2.1. Daugman’s Decidability Index

**Figure 6:**
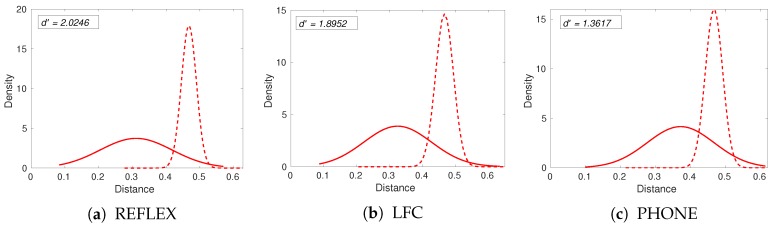
Normal distributions fitted to the normalized histograms of Hamming distances of matching (solid lines) and non-matching (dash lines) iris pairs are shown for three test image datasets.

Daugman’s decidability index [75] is a widely used method for assessing the performance of iris recognition systems [3,36,75]. In an iris recognition system like OSIRIS, a binary phase code is derived for each presented iris image. Then, the fractional Hamming distance to the phase code of a reference iris image is computed. The distributions of these Hamming distances are compared between a set of matching and a set of non-matching iris image pairs from a test dataset. The larger the overlap between the distributions, the more likely recognition errors become. The Daugman index ($d^{\prime }$) measures the separation of these distributions by

$$d^{\prime }=\frac{|\mu_{E}-\mu_{I}|}{\sqrt{\frac{1}{2}\left(\sigma_{E}^{2}+\sigma_{I}^{2}\right)}},$$

where $\mu_{E}$ and $\mu_{I}$ are the means and $\sigma_{E}$ and $\sigma_{I}$ are the standard deviations of the distributions. Larger values correspond to better discrimination. We follow this procedure using the $GC^{2}$ multi-modal biometric dataset and plot the histograms of the Hamming distances for the matching and the non-matching iris pairs in Figure 6. For visualization, normal distributions were fitted to the histograms.

**Figure 7:**
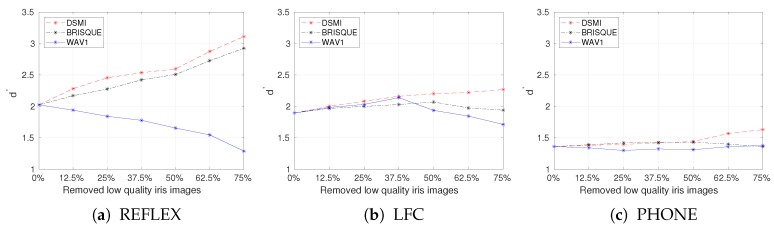
Daugman’s decidability index for all iris images, after filtering different parts of the iris images with the poorest quality using three image quality metrics on three test datasets.

We can now study the effect of quality filtering on the performance of the iris recognition system. In Figure 7, we show Daugman’s decidability index as a function of the fraction of removed poor-quality images. DSMI, BRISQUE, and WAV1 image quality metrics were used for quality filtering. Filtering out low-quality iris images using the DSMI metric leads to the largest performance improvement in the REFLEX dataset, while quality filtering in the PHONE dataset leads only to small improvements. This could be due to the DSMI metric performing better in quality assessment on iris images in the REFLEX dataset or to the PHONE dataset posing a greater challenge to the reference iris recognition system. The Daugman index for the PHONE dataset is only 1.36, compared to 2.02 and 1.90 for REFLEX and LFC, respectively (see Figure 6).

From the Daugman’s decidability index values in the three test datasets, as shown in Figure 7, we can conclude that filtering out the iris images with the poorest quality using the proposed DSMI metric improves the recognition accuracy of the reference iris recognition system. The BRISQUE metric also performs well in the REFLEX dataset, but it is not consistent for quality filtering in the LFC and PHONE datasets. WAV1 is not consistent with quality filtering on all three test datasets.

#### 5.2.2. Receiver Operating Characteristic Curve

The area under the curve (AUC) of the receiver operating characteristic (ROC) is a widely used performance metric for comparing the accuracy of iris recognition systems. The iris recognition system with the larger AUC is considered to be a more accurate system.

**Figure 8:**
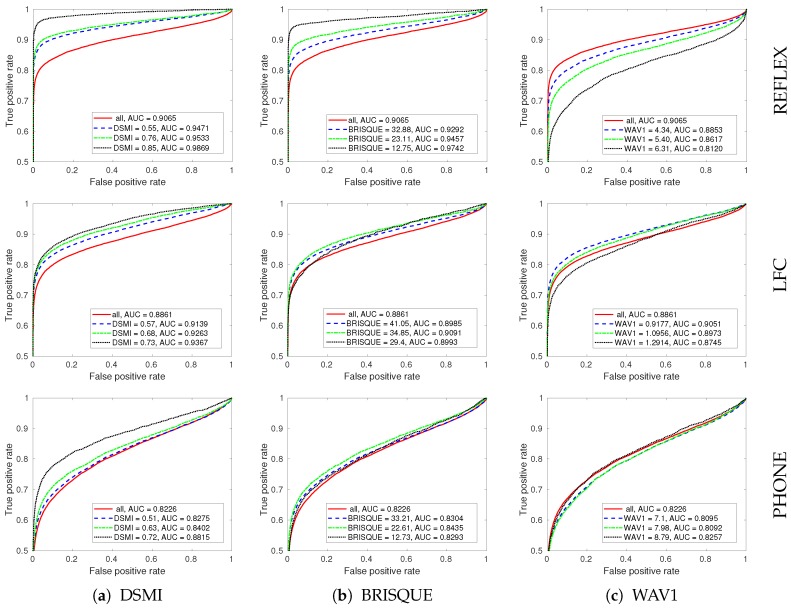
The receiver operating characteristic (ROC) curves for the three test datasets (REFLEX, LFC, and PHONE) with different quality filtering thresholds using our DSMI metric, BRISQUE, and WAV1. The solid red, dashed blue, dot-dashed green, and dotted black lines were plotted without quality filtering, after filtering out one-quarter, half, and three-quarters of the poorest-quality images, respectively.

To visualize and measure the improvements of the performance of the reference iris recognition system by filtering out the poor quality iris images, the ROC curves were generated for each dataset by plotting the true positive rate against the false positive rate at various fractional Hamming distances (see Figure 8).

Figure 8 shows the ROC curves for the three test datasets with different quality filtering thresholds using our DSMI metric, BRISQUE, and WAV1 metrics. The solid red lines in Figure 8 show the performance of the reference iris recognition system without quality filtering. Without quality filtering, the corresponding AUC value for the REFLEX dataset is 0.9065, for the LFC dataset it is 0.8861, and for the PHONE dataset it is 0.8226. The AUC values show again that the PHONE dataset is the most challenging one for the reference iris recognition system.

We also computed the AUC values after removing 1/4, 1/2, and 3/4 of the iris images with the poorest quality from each test dataset. The AUC values are listed in the figure legends for all of the test datasets. Using the proposed DSMI metric for quality filtering increased the AUC value in all test datasets.

In the REFLEX dataset, filtering out a quarter of the iris images with the poorest quality using the DSMI metric greatly improves the performance of the reference iris recognition system in terms of AUC by 0.0406 (4.5%). However, filtering out the second quarter only increases AUC by 0.0062 (0.65%). This indicates that the middle two quarters of the iris images have a small quality deviation, and filtering a part of these images does not result in a considerable improvement in the performance of the iris recognition system. However, filtering the third quarter of the iris images with the poorest quality improves the AUC significantly by 0.0336 (3.5%).

**Figure 9:**
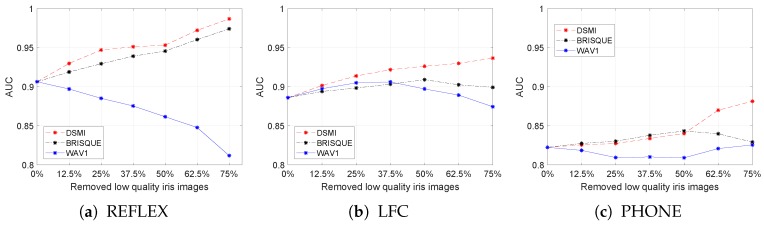
Area under the curve (AUC) values for all iris images after removing different parts of the iris images with the poorest quality.

The performance improvements for the LFC dataset after filtering out the first, second, and third quarters of the iris images with the poorest quality using the DSMI metric are 0.0278 (3.1%), 0.0124 (1.4%), and 0.0104 (1.1%), respectively. The values for performance improvement on the PHONE dataset are 0.0049 (0.6%), 0.0127 (1.5%), and 0.0413 (4.9%). Filtering out the first quarter of the iris images with the poorest quality using the DSMI metric only slightly improves the AUC value, but filtering out three quarters of the iris images with the poorest quality improves the performance significantly by 7.2%. We visualized these performance improvements in Figure 9.

The analysis of the AUC values shows that the performance of the reference iris recognition system has improved by quality filtering in all test datasets when using the DSMI metric for quality assessment. In contrast, BRISQUE is consistent for quality filtering for the REFLEX dataset, but not for the other two test datasets. WAV1 shows inconsistent performance in all test datasets.

The reason for this could be that the DSMI metric is optimized for assessing the image quality of iris images and BRISQUE for the perceptual quality of natural images. Both, however, can assess image quality for different image distortions. The WAV1 metric is optimized for blur assessment. Since blur is common in iris images taken with handheld devices, we compare our method with the WAV1 metric. However, the iris images in test datasets have more complicated authentic in-the-wild image distortions, and these distortions degrade the performance of WAV1 in all test datasets.

#### 5.2.3. Equal Error Rate

The equal error rate (EER) is the rate at which both accept and reject errors are equal. The EER is used for comparing the accuracy of classification systems with different receiver operating characteristic (ROC) curves. With the EER approach, the system with the lowest EER is considered the most accurate.

In Table 3, we calculated the EER values when three image quality metrics were used to filter out the poor-quality iris images from the test datasets. The greatest performance improvement is achieved by filtering out poor-quality iris images using the DSMI metric on the REFLEX dataset. The PHONE dataset is the more challenging dataset for the reference iris recognition system, resulting in higher EER values.

The results confirm that rejecting poor-quality images using the proposed DSMI metric improves the iris recognition performance consistently, while this observation does not hold for BRISQUE and WAV1 metrics.

In summary, for all of the test iris image datasets (REFLEX, LFC, PHONE) and all of the performance evaluation methods (Daugman’s decidability index, AUC, EER), the performance of the reference iris recognition system (OSIRIS, Version 4.1) increased consistently by filtering out iris images with the poor quality using the proposed DSMI quality metric. In contrast, for the other two image quality metrics (BRISQUE, WAV1), the experiments showed inconsistencies, i.e., removing more low-quality images did not always increase the performance of the reference iris recognition system.

**Figure 10:**
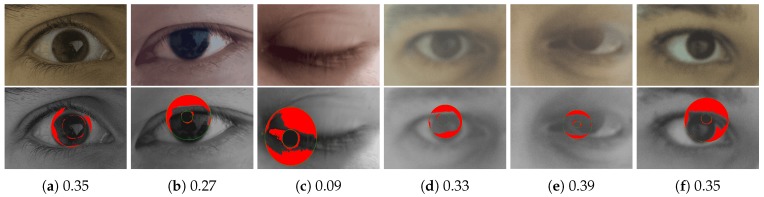
The first row shows some iris samples from the multi-modal biometric dataset $GC^{2}$ [36], which are classified as low-quality samples by our DSMI metric. All of these samples would be falsely rejected with high dissimilarity scores (>0.47) by the reference iris detection system. However, if we filter out a quarter of the iris images with the poorest quality from each test dataset, these samples will be removed and not passed to the iris recognition system. The second row shows the segmentation result of the segmentation module of the reference iris recognition system. The DSMI scores are listed below the iris samples.

Figure 10 shows some iris samples from the test datasets with poor quality scores predicted by the proposed DSMI metric. These samples will be filtered out when we remove a quarter of the iris images with the poorest quality from each test dataset. If we pass these samples to the reference iris detection system for iris recognition, all of them will be falsely rejected. Thus, the proposed DSMI metric can be used to decide whether an input iris sample should be enrolled in a dataset or rejected, and a new sample should be captured based on the quality score. Although our method is designed to consider only image covariates, some subject covariates, such as eyelid occlusion due to blinking, may also result in motion blur or other image quality distortions that can be measured by our proposed quality metric, as shown in Figure 10c. All iris samples shown in Figure 10 suffer from authentic image distortion and other quality degradation due to subject covariates.

**Figure 11:**
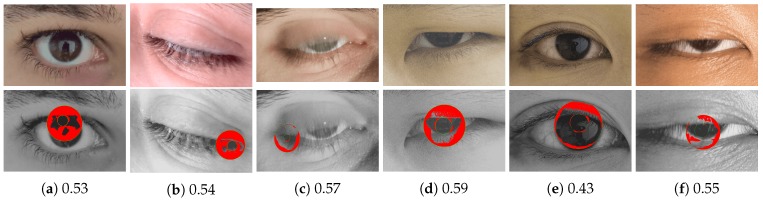
The first row shows some iris samples from the multi-modal biometric dataset $GC^{2}$ [36], which are classified by our DSMI metric as iris samples of sufficient quality if only one quarter of the iris images with the poorest quality are filtered out. Therefore, these images are passed to the iris recognition pipeline for further processing. However, all of these samples would be falsely rejected by the reference iris recognition system with high dissimilarity values (>0.47). The second row shows the segmentation result of the segmentation module of the reference iris recognition system. The DSMI scores are listed below the iris samples.

Figure 11 shows some iris samples with DSMI scores that are higher than the threshold for filtering out one quarter of the iris samples with the poorest quality from each test dataset. Our proposed framework passes these images for iris segmentation and identification when only a quarter of the iris images with the poorest quality are filtered out from the test datasets. However, all of these samples will be falsely rejected by the reference iris recognition system. Some of these images have quality degradation related to subject covariates, such as eyelashes obscuring the iris or closed eyes.

The iris samples that are shown in Figure 11 have fewer image distortions compared to the sample shown in Figure 10. Therefore, our quality metric predicts higher quality scores for these iris images. Some of these images have quality degradations related to subject covariates, such as eyelashes obscuring the iris or closed eyes. If we filter out half of the iris samples with the poorest quality, these samples will be filtered. However, by setting a higher quality filtering threshold, some iris samples may be rejected unnecessarily.

### 5.3. Computational Complexity

It is straightforward to assess the computational complexity of the DSMI quality metric by checking the algorithmic steps, outlined in Section 3.1, one by one. The result is a time complexity, linear in the size of the input image. More precisely, it is O(N×M×P), where N×M is the image size in pixels, and *P* is the number of points checked in the neighborhood of each pixel for deriving the sign and magnitude patterns.

We also recorded the actual speed of the quality metric using our implementation, running on an MSI GP60 laptop with an Intel Core i7 processor and 16GB RAM with MATLAB version 2018b in Ubuntu 18.04.3 LTS. We computed the DSMI quality scores on four parts of the test datasets, each containing iris images of the same size in pixels, ranging from 596×397 up to 2036×1358 (see Table 4). The table confirms the linear time complexity, amounting to roughly $0.06\times 10^{-6}$ seconds per pixel. At that processing speed, a throughput of 66 frames per second (FPS) can be achieved at resolution 596×397. For the higher resolutions, 625×537, 1233×810, and 2036×1358, the speed is 40, 16, and 6 FPS, respectively. Therefore, the proposed method can be used to assess the quality of iris images in interactive applications, such as iris recognition systems based on handheld imaging devices.

## 6. Conclusions and Future Work

In this paper, we presented a fast image quality metric, based on statistical features of the sign–magnitude transform to estimate the quality of iris images acquired by handheld devices in visible light. We suggest that this method can be used to decide whether an input iris sample should be enrolled in a dataset or rejected, and a new sample should be captured based on the quality score to improve the speed and the recognition rate of the reference iris recognition system.

We conducted extensive experiments to demonstrate these improvements using three performance methods for measuring the iris recognition accuracy on three large datasets acquired in unconstrained environments in visible light. The experiments showed that the proposed approach improved the accuracy of the reference iris recognition system.

However, we would like to point out that the inclusion of quality filtering in an iris recognition system can increase the computational costs of iris image recognition, and some iris images may be rejected unnecessarily. This could be caused by an error in the quality metric, by too conservative of a setting of the quality threshold, or by quality factors related to the subject covariates. In our future work, we will propose a metric for iris image quality assessment that takes into account all of these factors. Furthermore, another future work is to develop an algorithm to monitor criteria, such as recognition performance, time and number of photos required per person, and customer satisfaction, in order to dynamically adapt the threshold for quality filtering to achieve optimal performance.

It may also be promising to examine the use of the proposed quality metric to assess the quality of other biometric images, such as facial image, and NIR biometric images.

## Figures and Tables

**Table 1:** Summary of the artificially distorted iris image dataset.

**Reference Iris Images**					
**Degree of Iris Pigmentation**		**Number of Individuals**		**Number of All Iris Images**	
High		25		200	
Medium		25		200	
Low		25		200	
**Distorted Iris Images**					
**Distortion Type**	**MATLAB Function**		**Parameters Interval**	**Distorted Versions**	**All Distorted Iris Images**
GB	imgaussfilt(I, sigma)		0.5–5	10	6000
IN	imnoise(I,’salt &pepper’,density)		0.05–0.6	12	7200
OE	I+t		10–100	10	6000
MB	H=fspecial(’motion’,len, theta); imfilter(I,H,’replicate’)		10–60; 10–60	36	21,600
WGN	imnoise(I,’gaussian’,0,V)		0.002–0.02	10	6000
GB+WGN	imgaussfilt(I, sigma); imnoise(I,’gaussian’,0,V)		0.5–5; 0.002–0.02	100	60,000

**Table 2:** Summary of the $GC^{2}$ dataset.

Datasets	REFLEX	LFC	PHONE
Number of subjects	48	49	50
Total images	1422	1454	1379
Samples per eye	12–15	13–15	12–15
Matching pairs	9457	10,045	9092
Non-matching pairs	975,450	1,056,485	941,039
Camera	Canon D700	Light field camera	Phone Nexus
Lowest resolution	1085×724	327×218	450×300
Highest resolution	2813×1876	1080×1080	1811×1208

**Table 3:** The equal error rate (EER) values are calculated after filtering different parts of the iris images with the poorest quality from each test dataset. This table shows the EER values when all iris images are passed to the iris recognition system and after filtering out one quarter, half, and three quarters of the iris images with the poorest quality from the REFLEX, LFC, and PHONE datasets using the DSMI, BRISQUE, and WAV1 quality metrics.

Removed Part	REFLEX			LFC			PHONE		
	DSMI	BRISQUE	WAV1	DSMI	BRISQUE	WAV1	DSMI	BRISQUE	WAV1
0%	0.1469	0.1469	0.1469	0.1770	0.1770	0.1770	0.2466	0.2466	0.2466
25%	0.0987	0.1202	0.1714	0.1500	0.1604	0.1562	0.2418	0.2374	0.2594
50%	0.0878	0.978	0.1963	0.1376	0.1528	0.1692	0.2293	0.2276	0.2595
75%	0.0382	0.0520	0.2443	0.1287	0.1724	0.1955	0.1845	0.2412	0.2434

**Table 4:** Comparison of the average running time (seconds) on four sets of iris images with different resolutions.

Image Resolutions	596×397	625×537	1233×810	2036×1358
Average running time per image (seconds)	0.015	0.026	0.061	0.181
Average running time per pixel (microseconds)	0.065	0.062	0.062	0.064
Frames per second (FPS)	66	40	16	6
